# The Book World of Medicine and Science

**Published:** 1897-11-20

**Authors:** 


### THE BOOK WORLD OF MEDICINE AND SCIENCE.

SANITATION AND HEALTH. By Brigadier-General R. C. HART, V.C., C.B., R.E. Revised by Brigadier-Surgeon Lieut.-Colonel T. H. HENDLEY, C.I.E. Fourth edition. (London: William Clowes and Sons. 1897. Price 1s. 6d.)

It is not long since we commented on a previous edition of this little work. We note that it is "authorised by the War Office for use in all Army Schools," so that the fact of a new edition being called for may not mean very much. The original inspiration of the authors seems to have been derived from Parkes and Teale—two admirable sources. But much has happened since their works were written, and it might have been as well if Brigadier-Surgeon Lieut.-Colonel Hendley had kept a somewhat sharper eye upon the medical vagaries of his gallant comrade. However, the book will probably serve its purpose, notwithstanding the occasional grandiloquence of its diction. "The millionaire in his splendid room sees not the spectre of death rising from the basement and passing quietly through the costly carpet to beckon him to his grave" is certainly good, and ought to fetch Tommy Atkins.

SPINAL CARIES. By NOBLE SMITH, F.R.C.S.Edin., L R.C.P.Lond. Second edition. Pp. 153. Illustrations 89. (London: Smith, Elder, and Co. 1897. Price 5s.)

This is a good monograph on spinal caries by one who has had special experience of the disease and who has also made his experience available for others in a previous edition. The subject is treated mainly from a clinical point of view, although the pathology receives sufficient attention to illustrate and emphasise the various points which are so important for treatment. At the very outset the author draws attention to the great difficulty met with in diagnosing the early cases of this disease, a diagnosis which for successful treatment should be made as early as possible. Several interesting illustrations and cases are given of the possibility of lateral curvature and rotation of the spine being present in early caries and the harm that may be done by treating them by muscular exercises. A good description is given of the various diseases of the spinal column which may simulate caries, and their diagnosis is discussed; in cases of doubt he properly advises fixation of the spine, as the pain will be relieved even if malignant disease is present. The histories of several obscure cases are given in which either the disease was unrecognised during life or the symptoms were very uncertain; these are very interesting and beneficial reading. The latter half of the book is taken up with treatment. In place of the usual plaster of Paris or felt jacket, Mr. Noble Smith uses an adaptable metal splint invented by Mr. Chance. Most surgeons will admit the jacket is rather an unsatisfactory treatment for some cases of caries, and that deformity may even increase under its use. It seems to us that this metal splint should be more widely known and used than at present appears, to be the case; it is certainly cleaner and would appear to be quite as efficient. There cannot be two opinions as to the superiority of the author's splint for supporting the head over the usual jury mast arrangement. The illustrations from specimens in the various museums and those of the author's cases are very good. As regards Dr. Calot's method of forcibly straightening the spine, Mr. Smith thinks the period of six months too short for good repair to take place, and that in cases where new bone has been thrown out damage may be done by loosening of such new material, which would act as a foreign body. We note that "tubercular" and "tuberculous" are used indiscriminately; in a future edition we hope that "tuberculous" alone will be used. The book can be cordially recommended as a sound and reliable guide on spinal caries.

THE MYSTERY AND ROMANCE OF ALCHEMY AND PHARMACY. By C. J. S. THOMPSON. (London: The Scientific Press, Limited. 1897. 335 pages, illustrated. Price 5s.)

Mr. Thompson's patient researches into the history of alchemy and pharmacy have resulted in the elaboration of a work of considerable interest. It is clear that he has been confronted at every turn with the difficulty of marshalling his facts and observations in logical or chronological order. His method has been to deal more or less exhaustively with certain periods in the history of medicine, with certain masters and their followers, and with certain well-defined branches of the art. This has necessitated some slight degree of anticipation in the earlier and retrogression in the later pages of the work, and in some cases to a limited amount of repetition. In our opinion the author has chosen the better part—he has made his book a readable one, and each chapter in itself may be defined as a fairly complete story independent of what has gone before or what is to follow. For convenience sake the work has been divided into two parts. The first deals with the fathers of medicine, the wizards, the alchemists, the sorcerers, witches, &c., their history, their methods, and the results. The second part is entitled "Alchemy and Pharmacy in Literature," commencing with Chaucer and ending with Marryat. All the writers, with the exception of Goethe, Le Sage, and Dumas, are English, and include Spencer, Shakespeare, and Dickens. The earlier chapters in the second part are largely composed of such passages and references in the works of the authors dealt with as pertain to the various branches of medicine. The later chapters are rather a series of character sketches of the apothecary or "medico" typical of the time. The lessons to be learnt by the perusal are curiously instructive, and throw much light on latter-day methods. Quackery has existed from all times down to the present day; its methods have been and are the same, and the most successful men have often been those who have surrounded their operations with the least transparent veil of mystery. From time to time, however, honest men asserted themselves and obtained success; and the oath of Hippocrates, the Father of Medicine, which was exacted from every student on entering on his novitiate at his school, would at the present day do honour to any medical men who honestly fulfilled their vows. This oath is given *in extenso* on page 11 and onwards, and is worth the reading. We strongly advise all medical men who would know the history and traditions of their profession to give themselves a few hours pleasant relaxation in the perusal of Mr. Thompson's excellent work.

